# The Association between Health Insurance and All-Cause, Cardiovascular Disease, Cancer and Cause-Specific Mortality: A Prospective Cohort Study

**DOI:** 10.3390/ijerph17051525

**Published:** 2020-02-27

**Authors:** Liying Song, Yan Wang, Baodong Chen, Tan Yang, Weiliang Zhang, Yafeng Wang

**Affiliations:** 1 School of Economics and Finance, Xi’an Jiaotong University, Xi’an 710061, China; strong.11@stu.xjtu.edu.cn; 2 Mianyang Taxation Bureau of Sichuan Province, State Taxation Administration, Mianyang 621000, China; 3 Department of Accounting, School of Management, Xi’an Polytechnic University, No.19, Jinhua South Road, Xincheng District, Xi’an 710048, China; chen.baodong@stu.xjtu.edu.cn; 4 School of Finance and Accounting, Xi’an University of Technology, No. 58, Yanxiang Road, Yanta District, Xi’an 710054, China; yangtan@xaut.edu.cn; 5 School of Economics and Finance, Xi’an International Studies University, South Wenyuan Road, Chang’an District, Xi’an 710128, China; zhangwl@xisu.edu.cn; 6 Department of Epidemiology and Biostatistics, School of Health Sciences, Wuhan University, Wuhan 430071, China

**Keywords:** health insurance, mortality, cohort

## Abstract

The purpose of this study was to evaluate the association of insurance status with all-cause and cause-specific mortality. A total of 390,881 participants, aged 18–64 years and interviewed from 1997 to 2013 were eligible for a mortality follow-up in 31 December 2015. Cox proportional hazards models were used to calculate the hazards ratios (HR) and 95% confidence intervals (CI) to determine the association between insurance status and all-cause and cause-specific mortality. The sample group cumulatively aged 4.22 million years before their follow-ups, with a mean follow-up of 10.4 years, and a total of 22,852 all-cause deaths. In fully adjusted models, private insurance was significantly associated with a 17% decreased risk of mortality (HR = 0.83; 95% CI = 0.80–0.87), but public insurance was associated with a 21% increased risk of mortality (HR = 1.21; 95% CI = 1.15–1.27). Compared to noninsurance, private coverage was associated with about 21% lower CVD mortality risk (HR = 0.79, 95% CI = 0.70–0.89). In addition, public insurance was associated with increased mortality risk of kidney disease, diabetes and CLRD, compared with noninsurance, respectively. This study supports the current evidence for the relationship between private insurance and decreased mortality risk. In addition, our results show that public insurance is associated with an increased risk of mortality.

## 1. Introduction

In 2005, the World Health Assembly appealed to health systems to develop towards social health insurance and universal coverage [1]. In the most recent two decades, the percentage of those without health insurance decreased, and the percentage of those with public coverage increased among adults aged 18–64 in the United States [2]. Overall, in 2018, 13.0% lacked health insurance coverage, 19.7% were covered by public insurance plans, and 69.0% were covered by private health insurance plans [2]. The public focus on how policy changes increase health insurance coverage reflects a general assumption that insurance can improve health [3].

It is well established that health insurance is associated with increased access to medical care and helps protect against the high costs of catastrophic illness [4]. The previous study showed that individuals with insurance reported more physician visits and had a higher prevalence of recommended preventive services [5]. Private coverage has been demonstrated as being associated with lower all-cause mortality [3,6]. Additionally, one recent study also showed that publicly-financed health spending was associated with lower mortality rates in middle-income and low-income countries [7]. However, few studies evaluate the association of private coverage with cardiovascular disease (CVD), cancer and other cause-specific mortalities, including mortality from Alzheimer’s disease, kidney disease, influenza and pneumonia and chronic lower respiratory disease (CLRD). Additionally, among patients undergoing head and neck cancer surgery, patients with Medicaid/that were uninsured presented with more advanced tumors and had worse survival compared to the private insurance group [8]. Moreover, adults with public/other coverage had higher levels of psychological distress than those with private or no health insurance coverage [9]. Thus, it is not clear whether public/other coverage increases all-cause and cause-specific mortality.

Therefore, using a nationally representative sample of U.S. adults, we conducted a prospective cohort study to evaluate the association of insurance status (Uninsured, Public coverage and Private coverage) with all-cause, CVD, cancer and cause-specific mortality, adjusting for important socio-economic, behavioral, and health status factors. Subsequently, we also performed subgroup analysis based on the baseline characteristics for the association of insurance status with all-cause mortality, and sensitivity analyses were performed to assess the robustness of the results.

## 2. Materials and Methods

### 2.1. Study Design and Population

We used data from the publicly available files of the National Health Interview Survey. The NHIS is an annual national cross-sectional survey of the health status, nutritional status, and behaviors of the U.S. noninstitutionalized population administered by the National Center for Health Statistics [10,11]. The survey uses a multistage, probability-sampling approach to select participants from approximately 1900 geographically defined primary sampling units covering the 50 states each year. One adult and one child are randomly selected from each household for a detailed interview on their health and lifestyle behaviors. The annual response rate is approximately 80% of eligible households.

A total of 493,365 participants aged older than 18 years interviewed between 1997 and 2013 were eligible for a mortality follow-up on 31 December 2015. After excluding those aged older than 65 years and those missing data regarding their insurance status, 390,881 participants were included in final analysis. All survey participants provided informed consent prior to participating in the study. The approval of the Institutional Review Board was not required, since this study was based on secondary analyses of publicly available and deidentified data (https://www.cdc.gov/nchs/nhis/about_nhis.htm).

### 2.2. Outcomes

Using the ICD-10 codes, underlying causes of death were defined as follows: all-cause, CVD (I00-I09, I11, I13, I20-I51, I60-I69) including heart disease and stroke, cancer (C00-C97), chronic lower respiratory disease (CLRD) (J40-J47), Alzheimer’s disease (G30), diabetes mellitus (E10-E14), influenza and pneumonia (J09-J18), and kidney disease (N00-N07, N17-N19, N25-N27).

### 2.3. The Definition of Insurance Status

According to a series of questions, insurance status was divided into three groups: (1) no coverage; (2) private health insurance coverage; (3) public insurance coverage including insurance from Medicaid, non-Medicaid, Medicare, Children’s Health Insurance Program, a state-sponsored health plan, other government programs, or a military health plan. Adults with both private and other forms of health insurance coverage were included in the category with adults who had private coverage [12,13,14].

### 2.4. Covariates

Sociodemographic variables were measured at baseline, including sex, age, race (Hispanics, non-Hispanic white, non-Hispanic black, and others), educational attainment (less than high school graduate, high school graduate, and greater than high school graduate), and income level (low, middle, high). Health-related behavioral risk factors included smoking status (never smoked, former smoker, and current smoker) and physical activity (meeting the PA guidelines/ not meeting: Physical Activity Guidelines Advisory Committee [15]). Chronic diseases included BMI (<25.0, 25.0–29.9, and ≥30.0 kg/m^2^), hypertension (yes/no), diabetes (yes/no), stroke (yes/no), coronary heart disease (CHD) (yes/no), and cancer (yes/no).

### 2.5. Statistical Analysis

To describe baseline characteristics of study participants, we used weighted mean ± standard error and weighted percentage to display continuous variables and categorical variables, respectively. In addition, we examined the differences between the three categories of insurance status among participant characteristics by using an analysis of variance model for continuous variables and the chi-square test for categorical variables. Years of follow-up were calculated for each participant from the data of the starting point to the date of death or end of the study period (31 December 2015). We used cause-specific Cox proportional hazards models to calculate the hazards ratios (HR) and 95% confidence intervals (CI) to determine the association of insurance status with all-cause and cause-specific mortality [16]. In model 1, we adjusted age and sex. In model 2, we added income and education to the model 1 covariates. In model 3, we added BMI, smoking, drinking and physical activity to the model 2 covariates. In model 4, we added BMI, smoking, drinking and physical activity to the model 2 covariates. In model 5, we added hypertension, diabetes, stroke, CHD and cancer to the model 4 covariates. In model 6, we added self-rated health to the model 5 covariates. Furthermore, subgroup analyses were conducted to assess whether the association between insurance status and all-cause mortality varied among different baseline characteristics. Sensitivity analyses was also performed to assess the robustness of the results by excluding individuals with less than 2 years of follow-up or excluding participants with CHD or stroke or cancer. In addition, E-value was also calculated to evaluate the robustness of our results through estimating the minimum strength of association between any unmeasured confounder, insurance status, and all-cause mortality [17]. For the residual confounder to explain the observed association, the unmeasured risk should have a risk ratio associated with the exposure-outcome greater than the E value [18].

Sampling weights were used to account for the multistage sampling design. All statistical analyses were conducted using Stata13.0 (Stata Corp, College Station, TX, USA). All *p*-values refer to two-tailed tests and a two-sided *p* value of ≤0.05 was considered to be statistically significant.

## 3. Results

### 3.1. Baseline Characteristics

The baseline characteristics of the sample are displayed in Table 1. Of the 390,881 individuals included in the study, the weighted mean age was 40.0 years. There were 80,449 participants that were uninsured, 51,236 with public coverage and 259,196 with private coverage. Compared with persons who were uninsured, those with private coverage were more likely to be older, be female, be non-Hispanic White, have higher educational levels, have higher income, be never smokers, be current drinkers, meet adult physical activity guidelines, and have better self-rated health. Those with public coverage were more likely to be older, be female, be non-Hispanic, have higher educational levels, have lower income, have obesity, not meet adult physical activity guidelines, have more coexisting conditions and have worse self-rated health.

The sample group cumulatively aged 4.22 million years before their follow-ups, with a mean follow-up of 10.4 years; there were a total of 22,852 all-cause deaths with 7091 cancer deaths, 4010 CVD deaths, 3251 heart disease deaths, 759 stroke deaths, 882 CLRD deaths, 822 diabetes deaths, 289 influenza and pneumonia deaths, 377 kidney disease deaths, and 84 Alzheimer’s disease deaths, respectively.

### 3.2. Insurance Status and All-Cause Mortality

**Figure 1:**
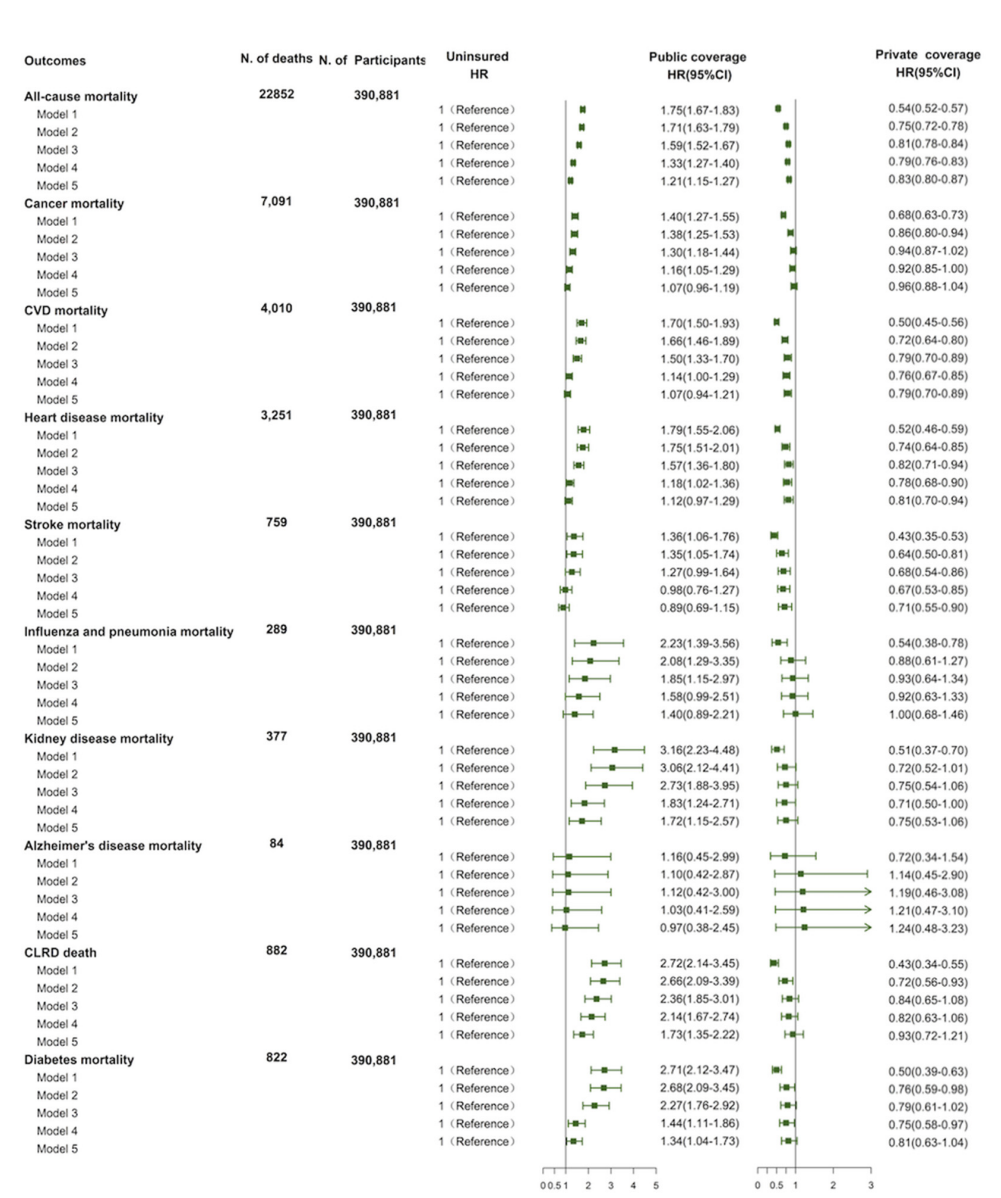
Risk of all-cause and cause-specific mortality in public insurance, and private insurance compared with uninsured. CIs: confidence intervals; CLRD: chronic lower respiratory disease; CVD: cardiovascular disease; HR: Hazard ratios.

In the model adjusted for age and sex, compared with uninsured individuals, privately insured persons have a 46% lower risk of all-cause mortality (HR = 0.54; 95% CI = 0.52–0.57), but those with public insurance have a 75% higher risk of all-cause mortality (HR = 1.75; 95% CI = 1.67–1.83; Table 2). In subsequent fully adjusted models, the effects are alleviated, private insurance was still significantly associated with a 17% decreased risk of mortality (HR = 0.83; 95% CI = 0.80–0.87), and public insurance was associated with a 21% increased risk of mortality (HR = 1.21; 95% CI = 1.15–1.27; Figure 1).

**Figure 2:**
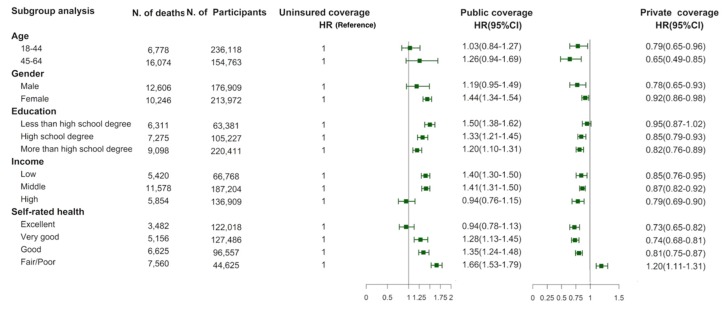
Stratified analysis of all-cause mortality risk by socioeconomic status and self-rated health. CIs: confidence intervals; HR: Hazard ratios.

The results of the subgroup analysis showed that the association between insurance status and all-cause mortality remained almost consistent in different age, gender, socioeconomic status and self-rated health groups. However, among those having fair/poor self-rated health, compared to uninsured individuals, those with private insurance had an elevated risk of all-cause mortality (HR = 1.20; 95% CI = 1.11–1.31; Figure 2). The magnitude of the impact of private insurance was slightly larger in participants that were 45-64 years old, males, with high education levels, high incomes, and that had excellent self-rated health.

### 3.3. Insurance Status and CVD, Cancer and Cause-Specific Mortality

After adjusting for age and sex, compared to individuals without insurance, those with private insurance had a reduced risk of mortality from CVD, cancer, heart disease, stroke, CLRD, diabetes, influenza and pneumonia, kidney disease and accidents (HRs from 0.43 to 0.54, All *p* < 0.05), but those with public insurance had an increased risk of mortality from these causes (HRs from 1.39 to 2.72, All *p* < 0.05) (Figure 1). In the full models, compared to noninsurance, private coverage was still associated with about 21% lower CVD mortality risk and the results were similar in magnitude for heart disease and stroke (CVD: HR = 0.79, 95% CI = 0.70–0.89; heart disease: HR = 0.81, 95% CI = 0.70–0.89; stroke: HR = 0.71, 95% CI = 0.55–0.90). However, public insurance did not increase the CVD mortality risk (CVD: HR = 1.07, 95% CI = 0.94–1.21; heart disease: HR = 1.12, 95% CI = 0.97–1.29; stroke: HR = 0.89, 95% CI = 0.69–1.15). In addition, public insurance was also inversely associated with the increased mortality risk of kidney disease (HR = 1.72, 95% CI = 1.12–2.57), diabetes (HR = 1.34, 95% CI = 1.04–1.73) and CLRD (HR = 1.73, 95% CI = 1.35–2.22), compared with noninsurance, respectively. Besides, we did not observe a significant association between insurance status and mortality risk of cancer, influenza and pneumonia disease, and Alzheimer’s disease (All *p* > 0.05).

### 3.4. Sensitivity Analyses

**Figure 3:**
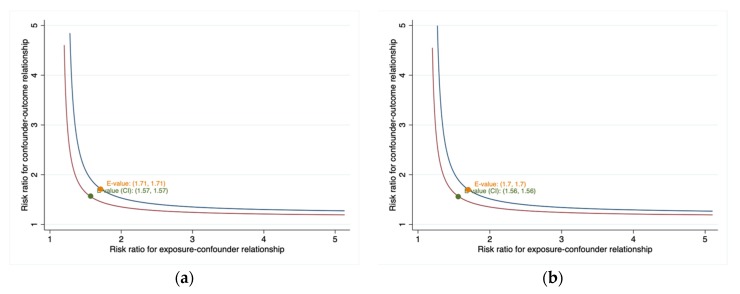
(**a**) Value of the joint minimum strength of association on the risk ratio scale that an unmeasured confounder must have with the exposure and the outcome to fully explain away an observed public coverage- all-cause mortality HR; (**b**) Value of the joint minimum strength of association on the risk ratio scale that an unmeasured confounder must have with the exposure and the outcome to fully explain away an observed private coverage - all-cause mortality HR.

Sensitivity analyses showed that almost all findings remained consistent for the associations observed in the full models after excluding participants with CHD, stroke and cancer at interview. Although the associations are attenuated by the exclusion of the first 2 years of follow-up, the HRs were not largely affected and remained significant (Table 2). In addition, the E-values for the point estimate for the association of public coverage (HR = 1.21) and private coverage (HR = 0.83) with all-cause mortality risk were 1.71 and 1.70, respectively, which indicated that the observed HR could be explained by an unmeasured confounder that was associated with both insurance status and all-cause mortality by an HR of 1.71 or 1.70 each, over and above the measured confounder (Figure 3). The corresponding CI could be moved to include the null by an unmeasured factor that was associated with both insurance status and all-cause mortality by an HR of at least 1.57 or 1.56 (Figure 3).

## 4. Discussion

This prospective study found that insurance status was robustly associated with mortality. Adjusted for sociodemographic, behavioral, and health status factors, private insurance was independently associated with about a 17% lower risk of overall mortality, but public insurance was associated with a 21% progressively higher hazard risk of total mortality, compared to individuals without any insurance. Furthermore, private insurance was associated with a 21% decreased risk of death from CVD, especially stroke. Participants with public insurance had a 32%–73% increased mortality risk of diabetes, kidney disease, and CLRD than uninsured persons. However, neither public insurance nor private insurance were associated with all-cancer mortality risk. In addition, we did not observe the association of insurance coverage with Alzheimer’s disease and influenza and pneumonia disease.

Many observational studies and one randomized controlled trial (RCT) have analyzed the association between insurance status and all-cause mortality [6,19,20,21,22,23]. Using data from the 1971–1975 National Health and Nutrition Examination Survey (NHANES), with mortality follow-ups through 1987, Franks et al. evaluated mortality among privately insured and uninsured individuals older than 25 years of age, controlling for demographic characteristics, behavior factors, and health status [21]. This study showed that private insurance coverage is associated with, significantly, a 20%-decreased risk of mortality (HR = 0.80; 95%CI = 0.65, 1.00). Using data from the 1988-1994 NHANES, with mortality follow-ups through 2000, Wilper et al. compared the mortality of privately insured and uninsured adults aged 17 to 64 years, adjusted for demographic characteristics, lifestyle factors, physician-rated health, and self-rated health, and found that privately insured was also associated with a lower mortality (HR = 0.71; 95%CI = 0.54, 0.94) [6]. In addition, Sorlie et al. analyzed data from the 1982–1985 Current Population Survey, with follow-up through 1987 and found that the relative risk for mortality associated with being privately insured was 0.83 for white women and 0.77 for white men [19]. In agreement with these results based population-based cohort data, our results based on data from the 1997–2013 NHIS, with mortality follow-up through 2015 also showed the similar results that hazard ratio of dying among the privately insured relative to the uninsured is 0.83 (0.80–0.87). To date, only one well-conducted RCT—the Oregon Health Insurance Experiment—has evaluated the effect of insurance on health outcomes and indicates that insurance may decrease mortality with an odds ratio of 0.84, which also supports our results [22,23]. However, our results are largely different from another study using data from the 1986-2000 NHIS, with mortality follow-ups through 2002. Although Kronick et al., found that being privately insured was associated with a decreased mortality HR of 0.91 (0.84–0.97), after controlling for demographic characteristics, body mass index, and smoking, the HR was attenuated to 0.97 (0.89–1.05) after additional adjustment for self-rated health and self-reported disability [19]. Recent studies suggested that the uninsured may cause individuals to underrate their health, perhaps due to distress or the inability to gain reassurance about minor symptoms [24,25]. In addition, adjustment for self-rated health and self-reported disability in a model may result in over adjustment, which may partly explain the attenuated estimate in Kronick’s study [26]. Moreover, more objective measures of baseline health were used in the study can lessen any such bias. 

Evidence on health insurance and mortality in middle- and low-income countries is limited [7]. Some retrospective cohort studies conducted in China showed that uninsured people have higher mortality than insured people [27,28]. However, some participants were with undergoing peritoneal dialysis or septic acute kidney injury [27,28]. Thus far, few prospective/retrospective cohort studies in general population are conducted in middle- and low-income countries. The previous study have showed that socioeconomic status is associated with differences in risk factors for mortality among high-income, middle-income, and low-income countries, which may suggest that the association between insurance status and mortality also differs among countries at varying economic levels [29]. In the future, well-designed cohort studies of the general population are required to explore associations between insurance status and mortality in countries of different socioeconomic levels, particularly among low- and middle -income countries.

Although private insurance was associated with decreased mortality risk, public insurance was associated with increased mortality risk. The mechanisms have been extensively studied for how health insurance affects mortality [4,30,31,32,33]. Previous studies have shown that socioeconomic factors, lifestyle factors, and getting care when needed played an important part in the mechanism [21,33]. Our results in Table 1 also observed that the privately insured participants had a higher education level, a higher income level, a lower proportion of smoking, a higher physical activity level, and a lower proportion of chronic diseases than uninsured participants [30]. However, compared with those with public insurance, the individuals with public insurance had a lower education level, a lower income level, a higher proportion of obesity, a higher proportion of physical inactivity, and a higher proportion of chronic diseases. The above reasons can partly explain the mechanisms through which insurance affects mortality. However, the mechanisms whereby health insurance affects CVD, diabetes, kidney disease, and CLRD mortality remain unclear. In the future, well-designed large prospective studies are needed to clarify the behavioral or social mechanisms involved.

This study has several strengths and limitations. Our study used a population-based prospective cohort design and large sample sizes from a nationally representative sample. The important variable of self-rated health can also be adjusted in this study. Besides, a new measure for confounding called E-value sensitivity analysis is also conducted to assess the potential effect of unmeasured confounders. The results from the E-value indicated that an unmeasured confounder associated with both insurance status and all-cause mortality by an HR of 1.71 or 1.70, respectively could move CI to include the null. The present study also has several limitations. Firstly, although the sample size is relatively large, this study is exploratory in nature and p-values are not adjusted for multiple test. Therefore, *p*-values may be overly significant for the association of insurance status and mortality. In addition, the stcrreg procedure, based on Fine and Gray’s proportional subhazards model is not supported by svy with vce (linearized) to account for complex survey designs. HR estimates in our study may be overestimated due to estimation bias caused by ignoring competing risks. Secondly, due to the nature of the observational study, we cannot exclude residual confounding, and this study design does not allow for causal inference and limits us to explore for the possibility of a two-directional relationship between health insurance status and all-cause and cause-specific mortality. Thirdly, the information of insurance status and other variables may change over this time period. The information at a single point in time was used in our study and the effect of gaining or losing coverage after the interview was unable to be measured which may underestimate or overestimate the association between insurance status and mortality. Fourthly, the present study only focused on participants 18-64 years, which limited our results as being generalized to persons ≥65 years.

## 5. Conclusions

In conclusion: our study found that private insurance was independently associated with about a 17% lower risk of total mortality, but public insurance was associated with a 21% increased total mortality risk compared to that of uninsured. In addition, private insurance was associated with a 21% decreased CVD mortality risk. Individuals with public insurance had a 32%–73% increased mortality risk of diabetes, kidney disease, and CLRD. In the future, carefully designed studies, such as randomized trials or mendelian randomization studies, are necessary to determine this association, as well as being warranted to clarify the biological, behavioral, and social mechanisms involved.

## Figures and Tables

**Table 1:** Baseline characteristics of participants, according to insurance status.

Characteristics	Uninsured	Public Coverage	Private Coverage	*p* Value
	No. (Weighted %)	No. (Weighted %)	No. (Weighted %)	
Total population	80449 (18.8)	51236 (10.9)	259196 (70.3)	
Age				<0.001
18–44	57215 (73)	28833 (57.9)	150070 (58)	
45–64	23234 (27)	22403 (42.1)	109126 (42)	
Gender				<0.001
Male	39779 (54)	17743 (40.6)	119387 (49.3)	
Female	40670 (46)	33493 (59.4)	139809 (50.7)	
Race				<0.001
Hispanic	30070 (29.9)	12079 (17.4)	32131 (8.9)	
Non-Hispanic White	33481 (50.5)	23147 (55.2)	180642 (76.1)	
Non-Hispanic Black	13419 (14.9)	13537 (22.3)	32279 (9.8)	
Non-Hispanic Other	3479 (4.8)	2473 (5)	14144 (5.2)	
Education				<0.001
Less than high school degree	26074 (29.8)	16532 (29.9)	20775 (7.6)	
High school degree	25216 (33.6)	15679 (32)	64332 (25.5)	
More than high school degree	28548 (35.8)	18617 (37.1)	173246 (66.6)	
Missing	611 (0.9)	408 (0.9)	843 (0.3)	
Income				<0.001
Low	25599 (26.8)	25471 (41.2)	15698 (4.7)	
Middle	47586 (61.7)	21568 (47.6)	118050 (43)	
High	7264 (11.5)	4197 (11.2)	125448 (52.4)	
BMI (kg/m^2^)				<0.001
<25	30780 (39.1)	16733 (33.5)	101702 (39.1)	
25–30	26613 (32.2)	15190 (29.4)	88102 (34.3)	
>30	19822 (24.7)	17321 (33.2)	60848 (23.4)	
Missing	3234 (4)	1992 (3.9)	8544 (3.2)	
Smoking status				<0.001
Never cigarette	43530 (52.6)	25328 (49.3)	155505 (60.1)	
Former cigarette	10180 (12.5)	8588 (17.4)	51300 (20.2)	
Current cigarette	26248 (34.2)	16980 (32.7)	50904 (19.1)	
Missing	491 (0.6)	340 (0.7)	1487 (0.6)	
Alcohol intake				<0.001
Lifetime abstainer	21399 (25.7)	15349 (30.1)	44236 (17.2)	
Former drinker	9970 (12.2)	10569 (20.3)	29185 (11.1)	
Current drinker	47652 (60.3)	24367 (47.8)	181845 (70.2)	
Missing	1428 (1.9)	951 (1.9)	3930 (1.5)	
Physical activity				<0.001
Meeting guideline	28973 (37.1)	14718 (30.1)	126613 (49.4)	
Not meeting guideline	49154 (59.8)	35138 (67)	124450 (47.3)	
Missing	2322 (3.2)	1380 (2.9)	8133 (3.3)	
Hypertension				<0.001
Yes	13390 (15.9)	17309 (32.4)	52292 (19.9)	
No	66926 (83.9)	33824 (67.4)	206705 (80.1)	
Missing	133 (0.2)	103 (0.2)	199 (0.1)	
Diabetes				<0.001
Yes	3555 (4.4)	6522 (12.1)	12566 (4.8)	
No	76831 (95.5)	44666 (87.8)	246481 (95.2)	
Missing	63 (0.1)	48 (0.1)	149 (0.1)	
CHD				<0.001
Yes	1111 (1.3)	2871 (5.4)	4527 (1.8)	
No	79271 (98.7)	48209 (94.6)	254488 (98.2)	
Missing	67 (0.1)	156 (0.1)	181 (0.1)	
Stroke				<0.001
Yes	840 (1)	2541 (4.7)	2182 (0.8)	
No	79564 (99)	48597 (95.1)	256874 (99.1)	
Missing	45 (0)	98 (0.2)	140 (0.1)	
Cancer				<0.001
Yes	2158 (2.7)	3495 (6.7)	12139 (4.7)	
No	78223 (97.3)	47649 (93.1)	246889 (95.2)	
Missing	68 (0.1)	92 (0.2)	168 (0.1)	
Self-rated health				<0.001
Excellent	21376 (27.7)	8606 (17.7)	92036 (36.6)	
Very good	23993 (30)	10480 (21.2)	93013 (35.7)	
Good	24453 (29.9)	14575 (28.7)	57529 (21.7)	
Fair/Poor	10576 (12.3)	17519 (32.2)	16530 (6)	
Missing	51 (0.1)	56 (0.1)	88 (0)	

Abbreviations: BMI, body mass index; CHD, coronary heart disease.

**Table 2:** Sensitivity analysis of the association of insurance status with all-cause and cause-specific mortality.

Outcomes	Uninsured	Public Coverage		Private Coverage	
	HR (95%CI)	HR (95%CI)	*p* Value	HR (95%CI)	*p* Value
Excluding participants with CHD, stroke or cancer					
All-cause mortality	1 (Reference)	1.24 (1.18,1.31)	<0.01	0.80 (0.76,0.84)	<0.01
Cancer mortality	1 (Reference)	1.08 (0.95,1.22)	0.23	0.88 (0.81,0.96)	0.01
CVD mortality	1 (Reference)	1.10 (0.95,1.27)	0.21	0.76 (0.67,0.87)	<0.01
Heart disease mortality	1 (Reference)	1.17 (0.99,1.38)	0.07	0.78 (0.67,0.91)	<0.01
Stroke mortality	1 (Reference)	0.87 (0.64,1.19)	0.38	0.69 (0.53,0.89)	<0.01
Influenza and pneumonia mortality	1 (Reference)	1.34 (0.84,2.14)	0.22	0.99 (0.64,1.52)	0.95
Kidney disease mortality	1 (Reference)	2.08 (1.24,3.49)	0.01	0.89 (0.60,1.33)	0.58
Alzheimer mortality	1 (Reference)	0.42 (0.13,1.36)	0.15	1.05 (0.38,2.90)	0.92
CLRD mortality	1 (Reference)	1.84 (1.38,2.46)	<0.01	0.89 (0.67,1.18)	0.43
Diabetes mortality	1 (Reference)	1.37 (1.01,1.86)	0.05	0.91 (0.69,1.20)	0.51
Excluding participants who died >2 year after the interview					
All-cause mortality	1 (Reference)	1.32 (1.26,1.39)	<0.01	0.85 (0.82,0.89)	<0.01
Cancer mortality	1 (Reference)	1.13 (1.02,1.25)	0.02	0.97 (0.90,1.06)	0.52
CVD mortality	1 (Reference)	1.25 (1.10,1.42)	<0.01	0.82 (0.73,0.93)	<0.01
Heart disease mortality	1 (Reference)	1.31 (1.14,1.51)	<0.01	0.85 (0.74,0.98)	0.02
Stroke mortality	1 (Reference)	1.02 (0.79,1.31)	0.90	0.73 (0.57,0.93)	<0.01
Influenza and pneumonia mortality	1 (Reference)	1.40 (0.91,2.14)	0.12	1.07 (0.70,1.62)	0.76
Kidney disease mortality	1 (Reference)	2.19 (1.48,3.24)	<0.01	0.79 (0.56,1.12)	0.19
Alzheimer mortality	1 (Reference)	0.99 (0.37,2.65)	0.99	1.24 (0.47,3.26)	0.67
CLRD mortality	1 (Reference)	1.77 (1.38,2.29)	<0.01	0.96 (0.74,1.24)	0.74
Diabetes mortality	1 (Reference)	1.58 (1.22,2.06)	<0.01	0.86 (0.66,1.11)	0.24

CHD, coronary heart disease; CI, confidence interval; CLRD: chronic lower respiratory disease; CVD, cardiovascular disease; HR, hazard ratio.
